# Comprehensive knowledge and HIV prevalence in two migrant mineworkers’ communities of origin in Gaza Province in Southern Mozambique: evidence from a cross-sectional survey

**DOI:** 10.11604/pamj.2021.40.19.22165

**Published:** 2021-09-07

**Authors:** Naira Jacira Luiz, Alda Ester Chongo, Paulino da Costa, Cynthia Semá Baltazar

**Affiliations:** 1 Department of Biological Sciences, Faculty of Science, Eduardo Mondlane University, Maputo, Mozambique,; 2 National Institute of Health, Maputo, Mozambique

**Keywords:** AIDS, behaviours, comprehensive knowledge, HIV, migrant, miners, prevalence, sexual behaviour

## Abstract

**Introduction:**

migrant mine workers are susceptible to engage in risky sexual behaviour due to their high mobility, putting at risk their families and home communities. Because comprehensive knowledge about HIV/AIDS is a key factor in reducing HIV infections, this study aims to understand the current state of knowledge about HIV in these communities, estimate HIV prevalence and evaluate the risk behaviour associated with comprehensive knowledge.

**Methods:**

secondary data analysis of a cross-sectional survey conducted in two communities of origin of mine workers in Gaza Province, targeting current and former mine workers of the South African mines and their relatives. Households were selected using simple random sampling methodology. Chi-squared tests and logistic regression analysis were used to assess statistical differences between comprehensive knowledge and categorical variables.

**Results:**

from a total of 1,012 participants, only 22.0% of the respondents had comprehensive knowledge about HIV. The overall HIV prevalence in these communities was 24.2% and the HIV prevalence in individuals with comprehensive knowledge was 18.6%. Among the respondents with comprehensive knowledge, 33.1% were male, 22.0% have worked in a South African mine and the median age was 34 years old. Individuals from Muzingane were almost twice as likely (AOR 1.7; 95% IC 1.21-7.44, p=0.014) to have less comprehensive knowledge about HIV than their counterparts in Patrice Lumumba.

**Conclusion:**

the results demonstrate a low level of comprehensive knowledge about HIV amongst this population and reveal an association between comprehensive knowledge about HIV and prevalence. Therefore, it is important to improve knowledge about HIV, its transmission and prevention amongst this population.

## Introduction

In 2018, UNAIDS estimated that Mozambique has 2.2 million people infected with HIV with only half currently taking ART [1]. In 2015, a population-based survey conducted in Mozambique estimated HIV prevalence at 13.2% in the adult population aged between 15-49 years old, ranking Mozambique as one of the most severely affected countries in the world by the HIV [1, 2]. Within Mozambique, Gaza is the province that contributes the most to such high HIV prevalence, with around a quarter of its adult population HIV-positive, totaling a prevalence of 24.4% [2]. Gaza province, in southern Mozambique, is characterized by high levels of cross-border migration, particularly men seeking work in the mines in South Africa and currently is the province with the largest number of mineworkers within the country [3-5]. Several studies have shown that migrant populations are more likely to engage in risky sexual behaviours which places them at risk for HIV not only themselves but also their families and communities [4, 6-8]. A Bio-behavioural Survey (BBS) was conducted in 2012 in Mozambique, among Mozambican mineworkers that worked in the South African mines showed that this population presents a high number of sexual partners, practice transactional sex with commercial sex workers, and low condom use with both their spouses and occasional partners. It also revealed an HIV prevalence of 22.3% amongst Mozambican mineworkers employed in the South African mines [3, 5]. Behavioural changes and the adoption of safer sexual habits, such as consistent condom use and reduction in the number of sexual partners, play a significant role in decreasing HIV transmission. Influenced by personal awareness on how HIV transmission can be prevented, especially targeting vulnerable populations, educational programs designed to increase comprehensive knowledge about HIV has become one of the main strategies to reduce HIV transmission worldwide [9-12]. HIV knowledge of HIV was defined by United Nations General Assembly Special Session on HIV/AIDS (UNGASS) in 2001, an indicator defined by the identification of the two primary ways of preventing the sexual transmission of HIV and the rejection of the most common misconceptions about HIV transmission [13, 14]. According to the Mozambique Survey of Indicators on Immunization, Malaria and HIV/AIDS (IMASIDA), conducted in 2015, comprehensive HIV knowledge amongst Mozambicans between the ages of 15 and 49 years is estimated at around 26% for men and 25% for women in rural areas and 26% in urban areas for both sexes. In Gaza province, the area in which this survey was conducted, comprehensive knowledge among adults is estimated at 28% [2]. This paper aims to investigate the association between comprehensive knowledge of HIV, HIV prevalence and risk behaviours in two migrant mineworkers communities of origin, in Gaza Province.

## Methods

### Study design and target population

This study is a result of a secondary analysis of data obtained from a cross-sectional survey that took place between May to June 2017 in two migrant mineworkers communities of origin located in Gaza province. The two communities selected were the rural Muzingane community and the urban Patrice Lumumba community. Both were chosen after a formative assessment that preceded the survey, which indicated that 60% of the households within these communities belonged to current or former mineworkers. The calculated sample size was approximately 1,000 participants correspondent to 250-300 households. It was calculated based on an HIV prevalence assumption of 24% and 95% significance level (power of 80% and a 10% margin for no response). The selection of households was systematic, through a random sampling methodology where a starting point was defined, and once the first house was identified from the defined central point the following house selected was the third one moving in a cardinal direction (n=3). Study participants include active and former mineworkers, their spouses and family members, as well as members of the community found in the household at the time of visit, aged 18 years or older, living in the communities of Muzingane or Patrice Lumumba, in Gaza Province, that were able to provide consent to participate in the study. Those who weren´t able to provide written informed consent by either the influence of alcohol or drugs, and those unable to speak Portuguese and/or Changana, were excluded.

### Data collection

A data entry tool was used to collect the data from a standardized questionnaire that comprised socio-demographic, behavioural and HIV and TB-related questions administered in either Portuguese or the local language, according to the participant´s preference. The questionnaire was deployed through the software Open Data Kit - ODK (Open Data Kit, version 2.0) and administered through a tablet and the collected information was stored on the ODK-Collect and synchronized with the central database through the ODK-Aggregate server. Through the survey, participants were asked socio-demographic questions such as age, educational level, marital status, if the person is a mineworker, and the nature of the relationship between respondent and mineworker. The inquiry also contained questions about sexual and risky behaviour, namely alcohol and drug consumption, sexual practices, condom use and the number of sexual partners. To evaluate the existence of comprehensive knowledge about HIV, the same standard set of knowledge questions used by IMASIDA (2015) were asked [2]. These included: 1) if a person can be protected from HIV by having only one non-infected sexual partner? 2) If a person can be protected from HIV by using condoms correctly and consistently? 3) If an apparently healthy person can be infected with HIV? 4) If a person can get infected by HIV by sharing a meal with an infected person? 5) Can HIV be transmitted through a mosquito bite? The HIV testing was based on the prevailing national sequential algorithm that uses two Rapid diagnostic tests, namely Determine^©^ HIV-1/2 (Alere Medical, Japan) and Uni-Gold™ HIV (Trinity Biotech, Ireland) [15]. Every participant received pre and post-test counselling, and the ones with positive results for HIV and/or TB were referred to a health care facility for follow up.

### Data analysis

The data collected was organized within an Excel database, and the tool used to proceed with the data analysis was the program RStudio. The variable “comprehensive knowledge about HIV” was transformed into a binominal variable, where the person able to answer all 5 questions related to HIV correctly was considered as a detainer of comprehensive knowledge about HIV. The independent variables were selected based on their relevance to justify the presence or absence of comprehensive knowledge, and variables that reflect risky sexual behaviours were also selected. The chi-squared test was applied to measure the association between comprehensive knowledge about HIV and HIV infection, considering all differences below 0.05 (p-value < 0.05) as statistically significant, and was again used to evaluate the association between risk behaviour and comprehensive knowledge about HIV. All the variables that were statistically significative (p-value < 0.05) moved on to the multivariable logistic regression model in order to identify the factors associated with comprehensive knowledge, using stepwise backward methodology.

### Ethical considerations

The study protocol was submitted and approved by both the Institutional Committee of Bioethics for Health (CIBS) at the National Institute of Health, and then by the National Committee of Bioethics for Health under the reference 041/CIBS-INS/2016. In order to participate in the survey, written informed consent was previously provided, and none of the participant´s personal identification details were requested, to protect their identities. All the staff that worked with the participants or had access to the information signed a confidentiality agreement previously.

## Results

### Socio-demographic characteristics of the study population

From a total of 1,026 eligible household members, 13 refused to participate in the survey, and one additional questionnaire was discarded during data cleaning. Thus, 1,012 respondents participated in the survey, of which most were females (75.2%) with a median age of 34 years. Almost half the population attended primary school (49.2%), and the most frequent religion was Protestant (45.1%). Over three-quarters of the participants stated that they have never worked on the mines (94%), and from the 27.7% participants that reported currently living with a mineworker or ex-worker, about half of them are spouses (54.5%) and the other half are relatives (44.8%) (Table 1).

**Table 1:** socio-demographic characteristics of the population from the two migrant mineworkers communities of origin in Gaza province, 2017 (N=1,012)

Characteristics	n	%	95% CI
Gender			
Female	761	75.2	72.4-77.8
Male	251	24.8	22.2-27.6
**Age range**			
18-24	295	29.2	26.4-32.1
25-39	375	37.1	34.1-40.1
40-49	150	14.8	12.7-17.2
+50	192	18.9	16.6-21.6
Median age (years)	34.1 (18/64)		
**Neighborhood**			
Muzingane	500	49.4	45.9-52.3
Patrice Lumumba	512	50.6	46.4-54.5
**Religion**			
Catholic	152	15.0	12.9-17.4
Protestant	419	41.5	38.4-44.5
Other	331	32.9	30.0-35.8
Islamic	5	0.5	0.1-1.2
None	102	10.1	8.3-12.1
**Marital status**			
Married/union	587	58.1	55.0-61.2
Divorced/separated	205	20.1	17.8-22.8
Single	220	21.8	19.3-24.4
**Has worked in a mine**			
Yes	59	6.0	4.5-7.5
No	953	94.0	92.5-95.5
**Currently lives in the same house with a miner or ex-miner former who works/worked in South Africa**			
**Yes**	278	27.4	24.8-30.4
**No**	732	72.3	69.6-75.2
**Relation with a miner or ex-miner who currently lives in the same house**			
Spouse	151	54.5	48.4-60.5
Sexual partner/boyfriend/girlfriend	2	0.7	0.13-2.9
Relative	124	44.8	38.8-50.8
**Level of education**			
Never been to school	103	10.2	8.4-12.3
Primary	495	49.2	46.0-52.3
Secondary	382	37.9	34.9-41.0
Greater than secondary	27	2.7	1.8-3.9

### HIV comprehensive knowledge and risk behaviours

Overall, 21.7% of the participants had comprehensive knowledge about HIV (data non shown in the table). Men (33.1%) and the respondents from P. Lumumba community (36.7%; 95% CI: 32.9-41.1) revealed to have a significantly higher knowledge about HIV (Table 2). Respondents who reported never consuming alcohol (24.1%; 95% CI: 20.9-27.7), never having sexual intercourse (27.3%; 95% CI: 11.6-50.4) and having more than one sexual partner (22.4%; 95% CI: 15.8-30.6) were found to have higher levels of HIV comprehensive knowledge. After analysing the chi-squared test results, it was found that HIV comprehensive knowledge is associated to both alcohol (p-value=0.05) and drug use (p-value=0.05), where the highest comprehensive knowledge was found amongst participants that never consumed alcohol (24.1%; 95% CI: 20.9-27.7), whereas knowledge was higher amongst those who are drug users (54.5%; 95% CI: 24.6-81.7) (Table 3).

**Table 2:** comprehensive knowledge about HIV among the population in two migrant mineworkers communities of origin in Gaza Province, 2017

Socio-demographic characteristics	Without comprehensive knowledge			With comprehensive knowledge			p-value
	n	%	IC	n	%	IC	
**Gender**							
Female	624	82.2	79.0-84.6	137	18.8	15.4-21.0	0.80
Male	168	66.9	60.7-72.6	83	33.1	27.4-39.3	
**Age range**							
18-24	221	74.9	69.5-79.7	74	25.1	20.3-30.5	0.59
25-39	295	78.7	74.1-82.6	80	21.3	17.4-26.0	
40-49	122	81.3	74.0-87.0	28	18.7	13.0-26.0	
Above 50	154	80.2	73.7-85.5	38	19.8	14.6-24.2	
**Neighborhood**							
Muzingane	468	96.6	91.0-95.5	32	6.4	4.5-9.0	0.001
Patrice Lumumba	324	63.3	58.9-67.4	188	36.7	32.9-41.1	
**Religion**							
Catholic	115	75.7	75.7-82.1	37	24.3	17.9-32.1	
Protestant	340	81.1	77.0-84.7	79	18.9	13.5-23.0	
Other	260	78.5	73.7-82.8	71	21.5	17.2-26.3	0.17
Islamic	40	80.0	74.1-85.9	1	20.0	10.7-29.3	
**Marital status**							
Single	176	80.0	74.0-85.0	44	20.0	15.0-26.0	
Married/union	456	77.1	74.1-81.0	131	22.3	19.1-26.0	0.77
Divorced/separated	200	77.9	76.1-86.1	45	22.1	13.8-24.0	
**Has worked in a mine**							
Yes	46	78	64.9-87.3	13	22.0	12.7-35.1	
No	746	78.3	75.5-80.8	207	21.7	19.2-24.5	0.30
**Currently lives in the same house with a miner or ex-miner former who works/worked in South Africa**							
Yes	213	76.6	71.1-81.4	65	23.4	18.6-28.9	
No	577	78.8	75.6-81.7	155	21.2	18.3-24.4	0.57
**Relation with a miner or ex-miner who currently lives in the same house**							
Sexual partner/boyfriend/girlfriend	2	100	19.9-100	0	0	0.0-80.2	0.73
Relative	97	78.2	69.7-84.9	27	21.8	15.1-30.3	
**Level of education**							
Never been to school	85	81.7	72.7-88.4	19	18.3	11.6-27.3	
Primary	381	77	73.0-80.6	114	23.0	22.7-31.4	0.13
Secondary	306	80.1	75.7-83.9	76	19.9	16.1-24.3	
Greater than secondary	19	70.4	49.6-85.5	8	29.6	14.5-50.3	

**Table 3:** relation between comprehensive knowledge about HIV and risky behaviours, in two migrant mineworkers communities of origin in Gaza Province, 2017

	Without comprehensive knowledge			With comprehensive knowledge			
Risky Behaviours	n	%	IC	n	%	IC	p-value
**Alcohol consumption**							
Consumes	198	81.5	75.9-86.0	45	18.5	14.0-24.1	0.05
Used to consume	113	83.7	76.1-89.3	22	16.3	10.7-23.9	
Never consume	481	75.9	72.3-79.1	153	24.1	20.9-27.7	
**Drug consumption**							
Consumes	5	45.5	18.1-75.4	6	54.5	24.6-81.7	0.05
Used to consume	17	85	61.3-96.0	3	15	39.7-38.7	
Never consume	769	78.5	75.7-80.9	212	21.5	49.6-88.4	
**Having sexual intercourse**							
Yes	773	78.3	75.1-20.3	220	21.7	19.6-24.9	0.74
No	16	72.7	49.7-88.4	6	27.3	11.6-50.4	
**Age of the first intercourse**							
Under 15 years	56	87.5	76.3-94.1	8	12.5	5.9-24.6	0.07
15-18	534	78.6	75.3-81.6	145	21.4	18.4-24.8	
19-25	129	78.2	71.0-84.1	36	21.8	15.9-29.0	
26-40	7	77.8	40.2-96.1	2	22.2	3.9-59.8	
Above 40	0	0	0.0-94.5	1	100	5.5-100	
**Circumstances of the first intercourse**							
Willingly	723	78.5	75.7-81.2	198	21.5	18.9-24.3	0.75
Unwillingly	8	72.7	39.3-92.7	3	27.3	7.3-60.7	
Under pressure	39	73.5	60.8-85.5	13	26.5	14.5-39.2	
**Has been forced to have intercourse**							
Yes	48	77.4	64.7-86.7	14	22.6	13.3-35.3	0.95
No	594	78.4	75.2-81.2	164	21.6	18.8-24.8	
**Has ever used a condom**							
Yes	482	79.3	75.9-82.4	126	20.7	17.6-24.2	0.63
No	290	76.7	72.1-80.8	88	23.3	19.2-27.9	
**Total of sexual partners for the last 12 months**							
One	535	78.4	75.1-81.4	147	21.6	18.6-24.9	0.98
Two or more	104	77.6	69.4-84.2	30	22.4	15.8-30.6	
**Use a condom with a sexual partner**							
Yes	409	80.5	76.7-83.8	99	19.5	16.2-23.3	0.15
No	339	76.9	72.6-80.7	102	23.1	19.3-27.4	
**HIV test result**							
Positive	204	83,3	77.9-87.6	41	16,7	12.4-22.3	0.02
Negative	588	76,7	73.5-79.6	179	23,3	20.4-26.5	

### HIV prevalence and comprehensive knowledge

The HIV prevalence found in both communities was 24.4% (data non shown in table), where the participants without comprehensive knowledge about HIV had an HIV prevalence three times greater than the ones with comprehensive knowledge (p-value < 0.05) (Table 3). The respondents from Muzingane were 1.7 (AOR=3.06; 95% CI: 1.24 - 7.44) times more likely to not have HIV comprehensive knowledge than the ones from P. Lumumba (Table 4).

**Table 4:** demographic and HIV risk predictors for having comprehensive knowledge about HIV among people from two migrant mineworkers communities of origin in Gaza Province, 2017

Socio-demographic characteristics	HIV comprehensive knowledge (N=220)					
	OR	95%CI	p-value	AOR	95%CI	p-value
**Gender**						
Male	**1.0**	**(0.7-1.5)**	**0.25**	**0.6**	**(0.26 -1.43)**	**0.29**
Female	Ref	-	-	ref	-	-
**Age range**						
18-24	Ref	-	-	ref	-	-
25-39	1.2	(0.8-1.7)	0.43	2.71	(1.09 - 7.13)	0.358
40-49	1.3	(0.8-2.1)	0.22	2.34	(0.70 - 7.59)	0.156
**50**	**1.1**	**(0.7-1.8)**	**0.56**	**2.62**	**(0.83 -8.27)**	**0.098**
**Neighborhood**						
Muzingane	**1.7**	**(1.2-2.3)**	**0.0007**	**3.06**	**(1.24 -7.44)**	**0.014**
Patrice Lumumba	Ref	-	-	ref	-	-
**Currently lives in the same house with a miner or ex-miner former who works/worked in South Africa**						
Yes	1	(0.7-1.4)	0.03	0.75	(0.35 -1.57)	0.45
No	Ref	-	-	ref	-	-
**Relation with a miner or ex-miner who currently lives in the same house**						
Spouse	1.2	(0.7-2.1)	0.5	-	-	-
Sexual partner/boyfriend/girlfriend	Ref	-	-	-	-	-
Relative	<0.005	(not determined)	1.0	-	-	-
**Alcohol consumption**						
Yes	0.9	(0.5-1.5)	0.58	0.70	(0.34 - 6.63)	0.350
No	Ref	-	-	-	-	-
**Having sexual intercourse**						
Yes	0.7	(0.3-2.1)	0.53	0.0003	(INF -1000)	0.986
No	Ref	-	-	-	-	-
**Has ever used a condom**						
Yes	-	-	Ref	ref	-	-
No	**1.2**	**(0.9-1.6)**	**0.3**	**0.99**	**(0.36 - 2.56)**	**0.985**
**Total of sexual partners for the last 12 months**						
One	Ref	-	-	-	-	-
Two or more	1.0	(0.7-1.6)	0.8	-	-	-
**HIV test result**						
Positive	1.1	(0.8-1.5)	0.7	-	-	-
Negative	Ref	-	-	-	-	-

## Discussion

In this survey conducted on residents of two migrant mineworkers communities of origin in Gaza Province, Mozambique, it was found that overall knowledge of HIV was low, in which only one in four persons living in these mining communities had comprehensive knowledge about HIV, whereby is within average according to a study that took place in three African countries and estimated comprehensive knowledge of HIV is below 50% in all the three study sites [10]. The participants from P. Lumumba had higher level of knowledge about HIV likely because this community is located in an urban environment that offers greater exposure to both social media and social interventions to reduce the impact of HIV infection [12].

Gender wise, 33.1% (83/251) of the participants with comprehensive knowledge on HIV were male, repeating the trend that was reported by the IMASIDA survey from, 2015 with a frequency of 31.2% for men versus 20.1% for women [2]. The reason behind this difference might be due to the fact that men are usually the decision-makers when it comes to sexual-life and they are generally more exposed to learning opportunities, while women tend to feel uncomfortable in discussing sex-related issues due to taboos that are usually associated with it [7]. Education level was revealed to also play a significant role on rates of comprehensive knowledge, where respondents with higher educational levels demonstrated more comprehensive knowledge, a trend that decreased as the educational levels also decreased, showing that education is a key factor to both perception and acquisition of knowledge about HIV [7].

Regarding risky behaviours, 24.1% (153/634) of respondents that reported never having consumed alcohol demonstrated greater rates of comprehensive knowledge. Alcohol consumption is considered high risk because of the behavioural changes that it may cause, leading to very unsafe sexual practices that increase the chances of the virus transmission [16].

From the total of 24.4% (245/1 012) respondents that tested HIV positive, 83.3% (204/245) didn´t have comprehensive knowledge about HIV, which based on the statistically significant association (p-value=0.029) between the variables, bespeaks that having comprehensive knowledge about HIV is associated to a lower prevalence of the virus. And even though comprehensive knowledge is not directly related to proactive approaches regarding sexual health, it is a key factor towards the adoption of better and safer sexual practices [17], including the reduction of sexual partners to one, avoiding alcohol and drug consumption as well as transactional and unprotected sex, as those may also increase the possibilities of infection by HIV.

## Conclusion

The results show a low-level of comprehensive knowledge about HIV as well as high HIV prevalence in the two migrant mineworkers communities of origin, where the highest HIV prevalence was reported on the group of participants that lacked comprehensive knowledge about HIV. These results provide key information to reverse the situation in which the mineworkers, their families and members of these communities of origin find themselves in, where public health strategies promoting awareness of HIV transmission, treatment and prevention can be improved, aiming towards the elimination of misunderstandings and misconceptions regarding HIV. It is also important to sensitize the populations about the importance of periodic testing, condom use and the reduction of sexual partners to one, as measures to reduce HIV infection and propagation.

### What is known about this topic

- The overall HIV prevalence in the migrant mineworkers communities of origin in Gaza, southern Mozambique is 24.2%;
- Gaza province is the one that contributes more to the high HIV prevalence of the country, and it is also a province with high level of cross-border migration.

### What this study adds

- This study revealed that members from the migrant mineworkers communities of origin in Mozambique have a low-level of comprehensive knowledge about HIV;
- Higher comprehensive knowledge was found in men, and in residents from the urban area;
- The respondents without comprehensive knowledge register a higher HIV prevalence when compared to the ones with comprehensive knowledge about HIV.
